# The National Provident Scheme

**Published:** 1921-09-17

**Authors:** Arthur Stanley, Alan G. Anderson, W. McAdam Eccles, J. F. Gordon Dill

**Affiliations:** Organising and Executive Committee of the National Provident Scheme. 3 Fenchurch Avenue, London, E. C. 3; Organising and Executive Committee of the National Provident Scheme. 3 Fenchurch Avenue, London, E. C. 3; Organising and Executive Committee of the National Provident Scheme. 3 Fenchurch Avenue, London, E. C. 3; Organising and Executive Committee of the National Provident Scheme. 3 Fenchurch Avenue, London, E. C. 3; Organising and Executive Committee of the National Provident Scheme. 3 Fenchurch Avenue, London, E. C. 3


### THE EDITOR'S LETTER-BOX.

**Correspondence on all subjects is invited, but we cannot in any way be responsible for the opinions expressed.**

### The National Provident Scheme.

*To the Editor of* The Hospital.

Sir,—It will doubtless be within the knowledge of your readers that certain of the large London hospitals have decided to offer their services to members of the National Provident Scheme for hospital and additional medical services.

We are now in a position to announce that the National Provident Scheme will definitely come into operation in London on November 1 next.

To those who are considering its adoption in other areas we have offered all the help and advice we can give, as we are assured that wherever the scheme attracts a sufficient membership the financial anxiety of the hospitals will be steadily lessened.

The scheme offers to those who come within certain prescribed limits of income, as well as to all those who are insured under the National Health Insurance Acts, practically all the higher resources of medicine, free of charge, beyond the payment of their annual subscriptions, and its advantages to the community are self-evident.

But there is a larger issue at stake. Men and women who are in receipt of small but regular incomes can, by joining the Provident Scheme, provide, during health, for the irregular and critical expense of illness. These persons do not ask for charity, and are not fit subjects for it; but when they are ill their income stops and they cannot afford the heavy costs of medical treatment outside the hospitals. They have consequently been forced hitherto to accept, in whole or in part, treatment provided by charity for the sick poor, or alternatively to go without.

The voluntary hospitals are now asking their patients to contribute towards their cost to the hospital, and members of the Provident Scheme will not only escape this inconvenient call upon them in illness, but have the satisfaction of knowing that they are paying their way, and that they will be helping the governing bodies of the several voluntary hospitals to maintain the high traditions and advantages of the voluntary system of hospital administration of which the nation is so justly proud.

The hospitals and other bodies co-operating in the scheme in London can supply to a limited number of members all the services promised, but as the success of the scheme will depend upon the satisfaction of its members, and as it is essential that the hospital and other medical and surgical benefits should be rendered promptly to members who require them, we have decided to limit strictly the number of members. Applications from those wishing to join the scheme will be dealt with in the order in which they are received until it is necessary to close the list.

We believe that the public are determined to save the voluntary system, and for this reason we launch the Provident Scheme in London with every confidence that it will be welcomed, and that its extension to other parts of the country is only a matter of time.—Yours faithfully,

(Signed) Arthur Stanley,
Dawson,
Alan G. Anderson,
W. McAdam Eccles,
J. F. Gordon Dill,
Organising and Executive Committee of the National Provident Scheme.

3 Fenchurch Avenue, London, E.C. 3,
September 7, 1921.

